# An outbreak of carbapenem-resistant OXA-48 – producing *Klebsiella pneumonia* associated to duodenoscopy

**DOI:** 10.1186/s13756-015-0049-4

**Published:** 2015-03-25

**Authors:** Axel Kola, Brar Piening, Ulrich-Frank Pape, Wilfried Veltzke-Schlieker, Martin Kaase, Christine Geffers, Bertram Wiedenmann, Petra Gastmeier

**Affiliations:** Institute of Hygiene and Environmental Medicine, Charité - University Medicine Berlin, Berlin, Germany; Medical Department, Division of Hepatology and Gastroenterology, Charité - University Medicine Berlin, Berlin, Germany; National Reference Laboratory for multidrug-resistant Gram-negative bacteria, Department for Medical Microbiology, Ruhr-University Bochum, Bochum, Germany

**Keywords:** *K. pneumoniae*, Carbapenemases, OXA-48, Outbreak, Endoscopy

## Abstract

**Background:**

Carbapenemase-producing Enterobacteriaceae (CPE) have become a major problem for healthcare systems worldwide. While the first reports from European hospitals described the introduction of CPE from endemic countries, there is now a growing number of reports describing outbreaks of CPE in European hospitals. Here we report an outbreak of Carbapenem-resistant *K. pneumoniae* in a German University hospital which was in part associated to duodenoscopy.

**Findings:**

Between December 6, 2012 and January 10, 2013, carbapenem-resistant *K. pneumoniae* (CRKP) was cultured from 12 patients staying on 4 different wards. The amplification of carbapenemase genes by multiplex PCR showed presence of the *bla*_OXA-48_ gene. Molecular typing confirmed the identity of all 12 isolates. Reviewing the medical records of CRKP cases revealed that there was a spatial relationship between 6 of the cases which were located on the same wards. The remaining 6 cases were all related to endoscopic retrograde cholangiopancreatography (ERCP) which was performed with the same duodenoscope. The outbreak ended after the endoscope was sent to the manufacturer for maintenance.

**Conclusions:**

Though the outbreak strain was also disseminated to patients who did not undergo ERCP and environmental sources or medical personnel also contributed to the outbreak, the gut of colonized patients is the main source for CPE. Therefore, accurate and stringent reprocessing of endoscopic instruments is extremely important, which is especially true for more complex instruments like the duodenoscope (TJF Q180V series) involved in the outbreak described here.

## Background

Carbapenemase-producing Enterobacteriaceae (CPE) are spreading worldwide, thereby increasing the problem of antimicrobial resistance for clinical and public health [1]. In healthcare facilities, CPE can cause serious infections and hospital outbreaks [2]. There are a few outbreak reports describing the association with duodenoscopy, [3-5] such as the recent outbreak of New Delhi metallo-ß-lactamase (NDM)-producing *Escherichia coli* transmitted by endoscopic retrograde cholangiopancreatography (ERCP) in Illinois, USA [6,7].

Here we report an outbreak of *Klebsiella pneumoniae* producing the oxacillinase-48 (OXA-48) carbapenemase which took place in Charité University Medicine, Berlin, a tertiary hospital with 139.000 hospital admissions a year.

Between December 6, 2012 and January 10, 2013, carbapenem-resistant *K. pneumoniae* (CRKP) was cultured from 5 patients staying on ward A. The patients were immediately transferred to single rooms or cohorted in shared rooms. Contact isolation precautions were taken for all 5 patients. Active surveillance screening for CRKP was introduced for all patients admitted to ward A and environmental samples from surfaces, medical devices, drugs, water, cleaning solutions, disinfectants etc. were taken. Neither the active surveillance nor the environmental samples revealed any further positive results for CRKP on ward A.

It was 4 weeks after the last CRKP was detected on ward A, when another 5 cases of CRKP emerged on different locations of the hospital: On ward B, there were 3 patients and on wards C and D each one further patient with CRKP-positive results.

## Methods

The medical records of patients with CRKP were reviewed. As four patients (one from ward A, one from ward B and the two single patients from wards C and D) underwent duodenoscopy for ERCP, the endoscopy records were also inspected.

From December 10, 2012 (after the second patient had been tested positive for CRKP) Patients who were admitted to wards A and B or underwent duodenoscopy using one specific instrument (TJF Q180V series) were screened for rectal CRKP colonization. In addition, the environment and all duodenoscopes of the endoscopy unit were sampled.

CRKP screening of patients was performed by plating rectal swabs on selective culture media containing cefpodoxime (ChromID ESBL, bioMerieux) and on MacConkey plates on which an ertapenem disk (10 μg) was placed.

Swabs taken for environmental sampling were enriched in trypticase soy broth (TSB) for 7 days at 37°C. Liquid samples were filtered through 0.2 μm cellulose membrane filters which were enriched in TSB for 7 days at 37°C. Disinfectants and soaps (1 mL) were transferred to TSB containing neutralizers (3% Tween 80, 3% saponin, 0.1% histidin, 0.1% cystein). Subsequently, the enriched TSB samples were cultured on Columbia and MacConkey plates.

Duodenoscopes were sampled by flushing each channel with 20 ml of sterile saline solution and swabbing the ends of the channels. A 10 mL sample of the flushing solution was neutralized and filtered. Filters and swabs were processed as described above.

Species identification and susceptibility testing was done using a VITEK 2 system.

For detection of carbapenemase genes, a multiplex polymerase chain reaction (PCR) containing primers for *bla*_OXA-48_, *bla*_BIC_, *bla*_NDM_ and *bla*_KPC_ was performed [8].

Strain typing of CRKP was done by comparison of XbaI macro restriction profiles generated by pulsed field gel electrophoresis (PFGE) according to the criteria of Tenover [9].

## Results

**Figure 1:**
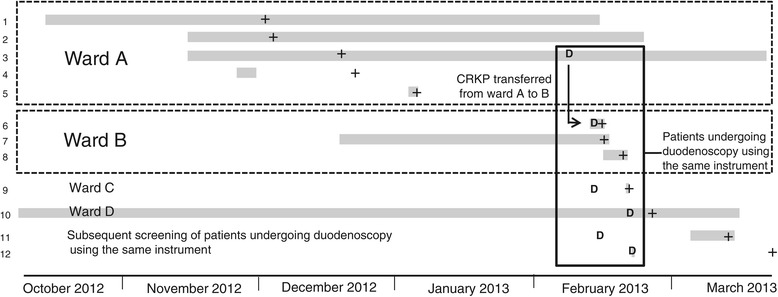
**Description of the CRKP outbreak and its association to duodenoscopy.** Legend: 1–12: Case number; Grey bars: Duration of hospital stay; +: Isolation of CRKP; D: Duodenoscopy.

An overview of the spatial and temporal relationship and the characteristics of the patients is given in Figure 1 and Table 1, respectively.

**Table 1:** **Characteristics of CRKP cases**

**Patient**	**Gender**	**Age (years)**	**Date of positive CRKP sample**	**Source**	**Infection/colonization**	**Ward**	**Date of duodenoscopy**
1	m	71	06/12/12	Tracheobronchial secretion	Respiratory tract infection	A	-
2	m	20	10/12/12	Rectal swab	Septicemia	A	-
3	m	25	27/12/12	Rectal swab	Septicemia	A	08/02/13
4	f	34	30/12/12	Blood culture	Septicemia	A	-
5	f	72	10/01/13	Rectal swab	Colonization	A	-
6	m	58	13/02/13	Blood culture	Septicemia	B	12/02/13
7	f	32	14/02/13	Intra-abdominal swab	Septicemia	B	-
8	m	57	18/02/13	Rectal swab	Colonization	B	-
9	m	26	18/02/13	Rectal swab	Surgical site infection	C	12/02/13
10	m	39	26/02/13	Rectal swab	Respiratory tract infection	D	20/02/12
11	m	43	14/03/13	Rectal swab	Septicemia	E	13/02/13
12	f	61	24/03/13	Rectal swab	Colonization	F	20/02/13

Reviewing the endoscopy records of the four CRKP positive patients who underwent ERCP revealed that the same duodenoscope had been used in all of them. The flushing solutions and swabs from the respective duodenoscope grew no additional CRKP, nor did the samples from the other duodenoscopes or the environment of the endoscopy unit. Only enterococci were cultured from the flushing solution of one of the other duodenoscopes.

The review of the endoscope records identified 22 additional patients who underwent ERCP with the respective duodenoscope, of which 19 were available for rectal screening. From two of these patients, CRKP were recovered from the rectal swabs.

Typing of the 12 CRKP strains (5 from ward A, 3 from ward B and the 4 single patients who underwent ERCP) revealed that they were closely related (less than 3 bands difference in PFGE). The amplification of carbapenemase genes by multiplex PCR showed presence of the *bla*_OXA-48_ gene.

Apart from the ERCP procedure using the same duodenoscope, the review of the medical records did not develop any further linkage between the patients from wards A and B and the four single CRKP cases.

## Discussion

In our hypothesis, CRKP were first introduced to ward A by patient 1 as part of the patient’s gut flora and then transmitted to patients 2 – 5 on ward A, most probably by contact (either by the hands of staff members or by contaminated medical devices or surfaces). Subsequently, one of these patients (patient 3) underwent ERCP, which led to contamination of the duodenoscope and the transmission of CRKP to patient 6, who acted as the source of transmission of CRKP to the patients 7 and 8 on ward B. The contaminated duodenoscope is also thought to have transmitted CRKP to four additional patients (patients 9 to 12), who all underwent ERCP using the same endoscope.

Although culturing of the duodenoscope did not recover CRKP, this does not exclude its association with CRKP transmission. While recommended for surveillance testing of endoscope reprocessing in the German guidelines, [10] flushing of the instrument channels with NaCl may not be sensitive enough to discover endoscope contamination [11] – particularly after the instrument was reprocessed several times before sampling such as in this case.

Additionally brushing the endoscope channels might have been the method of choice to prove contamination with the respective outbreak strain of CRKP [12], but the instrument was sent to the manufacturer for maintenance before it could be probed again.

Nevertheless, there are several epidemiological hints pointing to the duodenoscope as source of the CRKP-transmission: i) duodenoscopy using the instrument in question was the only epidemiological link between patients from different wards ii) following duodenoscopy, CRKP were only cultured if the implicated instrument was used iii) after the instrument in question was sent for maintenance (which revealed defects of the external layers and the distal cap of the respective duodenoscope), no further CRKP were cultured.

Reviewing the complete reprocessing operation in our endoscopy unit, we could not identify any deviations from the procedures recommended by the manufacturer of the duodenoscope. However, in one instance enterococci were cultured from one of the reprocessed duodenoscopes, which are indicative for insufficient cleaning and disinfection [10]. Therefore, the reprocessing procedures were not in every case sufficient, most probably due to the complex physical design of the endoscope’s distal end which complicated reprocessing as additional manual steps had to be performed strictly complying with the manufacturer’s advice: The distal cap of the duodenoscope was not removable and required accurate manual brushing and locking of the forceps elevator at 45° before automated reprocessing could be done.

Recently, an outbreak of NDM-producing *E. coli* associated with ERCP which ended after the duodenoscopes were sterilized with ethylene oxide was published [6,7]. The authors concluded that ERCP-related transmission should be considered in case of an outbreak with CPE, as the complex design of duodenoscopes “might pose a particular challenge for cleaning and disinfection” [6] and makes cleaning difficult. This conclusion is supported by a recent review which identified gastrointestinal endoscopy to be a risk factor for infection and colonization with CPE [13]. The complex physical design of the endoscope’s distal end could also have contributed to the outbreak described here, as it required strict adherence to the procedures recommended by the manufacturer. In a recent safety communication the U.S. Food And Drug Administration states that the transmission of microorganisms may even occur when manufacturer reprocessing instructions are followed correctly and, therefore, is continuing to evaluate information about documented and potential infections due to duodenoscopic instruments. In the meantime, patients should be informed about the potential risks of duodenoscopy including infections due to transmission [14].

Though the outbreak strain was also disseminated to patients who did not undergo ERCP and environmental sources or medical personnel contributed to the outbreak on wards A and B, the gut of colonized patients is the main source for CPE. Therefore, accurate and stringent reprocessing of endoscopic instruments is extremely important to prevent the transmission of multidrug resistant organisms or blood borne viruses – in particular, when more complex endoscopic instruments are involved as in the outbreak described here.

## Abbreviations

CPE: Carbapenemase-producing Enterobacteriaceae
CRKP: Carbapenem-resistant *K. pneumonia*
ERCP: Endoscopic retrograde cholangiopancreatography
NDM: New Delhi metallo-ß-lactamase
OXA-48: Oxacillinase-48
PCR: Polymerase chain reaction
PFGE: Pulsed field gel electrophoresis
TSB: Trypticase soy broth
